# Graphitic platform for self-catalysed InAs nanowires growth by molecular beam epitaxy

**DOI:** 10.1186/1556-276X-9-321

**Published:** 2014-06-25

**Authors:** Qian D Zhuang, Ezekiel A Anyebe, Ana M Sanchez, Mohana K Rajpalke, Tim D Veal, Alexander Zhukov, Benjamin J Robinson, Frazer Anderson, Oleg Kolosov, Vladimir Fal’ko

**Affiliations:** 1 Physics Department, Lancaster University, Lancaster LA1 4YB, UK; 2 Department of Physics, University of Warwick, Coventry CV4 7AL, UK; 3 Stephenson Institute for Renewable Energy and Department of Physics, University of Liverpool, Liverpool L69 7ZF, UK; 4 Department of Physics and Astronomy, University of Manchester, Manchester M13 9PL, UK; 5 Oxford Instruments, Tubney Woods, Abingdon OX13 5QX, UK

**Keywords:** Nanowires, Graphite, Molecular beam epitaxy

## Abstract

We report the self-catalysed growth of InAs nanowires (NWs) on graphite thin films using molecular beam epitaxy via a droplet-assisted technique. Through optimising metal droplets, we obtained vertically aligned InAs NWs with highly uniform diameter along their entire length. In comparison with conventional InAs NWs grown on Si (111), the graphite surface led to significant effects on the NWs geometry grown on it, i.e. larger diameter, shorter length with lower number density, which were ascribed to the absence of dangling bonds on the graphite surface. The axial growth rate of the NWs has a strong dependence on growth time, which increases quickly in the beginning then slows down after the NWs reach a length of approximately 0.8 μm. This is attributed to the combined axial growth contributions from the surface impingement and sidewall impingement together with the desorption of adatoms during the diffusion. The growth of InAs NWs on graphite was proposed following a vapour-solid mechanism. High-resolution transmission electron microscopy reveals that the NW has a mixture of pure zinc-blende and wurtzite insertions.

## Background

During the last few years, there have been increasing efforts in developing growth of functional hybrid structures of III-V semiconductors on graphene or graphite thin films. In these hybrid structures, the graphene (or graphite) could function as a device electrode owing to its excellent optical transparency, electrical conductivity and flexibility [1]. Also, because of its two dimensional (2D) crystal structure and the chemical stability, the graphene serves as a platform for growth of semiconductors via van der Waals epitaxy. A few semiconductor materials on graphene have been obtained including nanowires (NWs) of InAs [2,3] and InGaAs [4,5] grown by metal-organic chemical vapour deposition (MOCVD), GaAs [6] NWs grown by molecular beam epitaxy (MBE), ZnO NWs [7,8], as well as thin films such as GaN on graphite substrates via an intermediate ZnO layer [9]. In particular, NWs on graphene hybrid structures are of great interest due to the intriguing properties of NWs, including the capacity of dislocation-free growth in lattice-mismatched epitaxy [10-12], efficient light absorption and emission [13,14], freedom of composition integration and reduced materials consumption. NW devices on Si have been demonstrated such as lasers [15], light-emitting diodes [16] and photovoltaic solar cells [17-19]. Consequently, epitaxial NWs on mechanically flexible and electrically conductive graphene or graphite hold great potential in fabricating cost-effective and flexible devices.

Of particular interest are the hybrid structures of InAs NWs on graphite, which may have a number of device applications such as infrared light emitters, photodetectors and thermophotovoltaic electricity generation. Although InAs NWs have been obtained by MBE on Si [20-22], InAs (111)B [23], GaAs (111) [24] and InP (111) [25], InAs NWs on graphene/graphite have only been obtained by MOCVD [2-5]. MBE as a well-developed epitaxy technique has advantages of low growth temperature and precise control of growth thickness and composition. In this paper, we report the realisation of InAs NWs on graphite by MBE via a droplet-assisted technique. Due to the lack of surface bonds of graphite, initial nucleation for epitaxial growth is challenging which generally requires pre-growth treatment, e.g. oxygen reactive ion etching treatment onto the graphite thin film was required [3]. In our MBE growth, the metal droplets act as seeding for nucleation to initiate the growth of NWs. This technique provides freedom in controlling the size and density of the resulting NWs. It also removes the need of pre-growth treatment.

## Methods

The InAs NW samples were grown on a solid-source MBE system. The graphite films were mechanically exfoliated from highly oriented pyrolytic graphite (HOPG) and transferred onto chemically cleaned Si (111) substrates (10% HF solution for 2 min). The substrates were loaded into the system and outgassed at 650°C for >5 h. The growth started from an indium droplet deposition at pre-optimised growth conditions under a background pressure of approximately 10^−9^ mbar, then the substrates were heated up to temperatures of 450°C to 500°C followed by spontaneous opening of In and As for NWs growth. As_4_ was used for the growth at a beam equivalent pressure (BEP) of approximately 10^−6^ mbar. In order to understand the growth mechanisms, a series of samples were grown for different times, and a sample of InAs NWs on bare Si (111) substrate was also grown at identical growth conditions. The Si substrate was chemically cleaned by 10% HF solutions for 2 min to remove the native oxide. The geometry and the crystalline quality of the resulting NWs were examined by scanning electron microscopy (SEM) and high-resolution transmission electron microscopy (TEM). The indium droplet deposition was calibrated in terms of growth rate, deposition thickness and growth temperature by growing a series of samples at various temperatures of 145°C to 310°C using In-flux in the range of 2.2 to 6.0 × 10^−7^ mbar.

## Results and discussion

**Figure 1:**
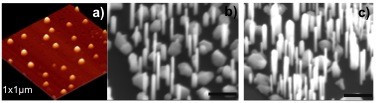
**AFM image of pre-calibrated In droplets and SEM image of grown InAs NWs.** A 1 × 1 μm AFM image of pre-calibrated indium droplets grown at optimal conditions **(a)** and 45°-tilted SEM image of InAs NWs grown for 20 min on **(b)** graphite and Si (111) **(c)**. The scale bar is 400 nm.

Figure 1a is the atomic force microscope (AFM) image of optimal sample showing that the droplets have an average diameter of approximately 70 nm, height of approximately 20 nm and density of approximately 6 × 10^8^ cm^−2^. We found that 3 ML indium deposition grown at 220° with a growth rate of 0.01 ML/s gives uniform droplets suitable for NWs' growth. Figure 1b shows the 45°-tilted SEM image of InAs NWs grown on HOPG for 20 min. All the NWs are vertically aligned on the surface without tapering, i.e. highly uniform diameter along the entire length. The NWs also have a homogeneous diameter distribution with a hexagonal cross-section, and no metal droplets are present on the top of the NWs. The average diameter, length and number density of the NWs are 78 ± 5 nm, 0.82 ± 0.28 μm and approximately 4 × 10^8^ cm^−2^ respectively. The SEM image also shows that parasitic InAs islands were formed on the surface during growth. Based on an estimate from large-area SEM images, the InAs islands cover 38% of the surface. As the areal coverage of NWs is approximately 2%, almost 60% of the surface remains bare. As growths on graphite without indium droplets led to NWs with a density one order of magnitude lower than that with droplets, we assume that droplets activate the growth of NWs.

The vertical alignment of the NWs is due to the low surface energy along the (111) orientation. The morphological parameters of the resulting NWs are similar to those of GaAs NWs on graphite by MBE [6]. However, in comparison with MOCVD grown InAs NWs on graphite (diameter of approximately 42 nm [2] and 30 nm [4] with a density of 6 to 7 × 10^8^ cm^−2^), our MBE-grown InAs NWs are doubled in diameter with half the density. This is probably because of the non-requirement of activation and dissociation at the surface during the growth in MBE leading to longer surface diffusion of the adatoms, resulting in larger diameter and lower density [26]. In addition, the absence of surface dangling bonds on the graphite surface gives rise to van der Waals epitaxy which is proposed to be different from general Frank-van der Merwe growth mode in MBE (layer-by-layer growth). In order to understand this effect, a few samples of InAs NWs were grown on Si (111) under identical growth conditions. These led to repeatable NWs as shown in SEM image (Figure 1c) for typical resulting NWs. It shows that the NWs on Si have an average diameter, length and number density of 65.0 ± 2.2 nm, 1.1 ± 0.3 μm and 1.2 × 10^9^ cm^−2^ respectively, which are thinner and longer with higher number density. The observed geometrical difference between the NWs grown on graphite and on Si could be attributed to the suppression of adatom diffusion. The typical diffusion-induced growth mode in MBE-grown NWs is dictated mainly by the diffusion of adatom from the side facets to the droplet but not by the adsorption on the drop [27]. Consequently, a modification to the diffusion of adatoms by different substrates will lead to significant variations in both axial and radial NWs growths. The area coverage of parasitic islands is approximately 58% which is higher than that on graphite (38%). These differences are further evidence that the weak surface bonds of graphite favour adatom diffusion.

The absence of metal droplets on the top of NWs is similar to the InAs NWs grown on Si by MBE which was ascribed to vapour-solid (VS) growth mechanism [20-22]. As the growth conditions of our NWs are similar, we assume that our NW growth also follows a VS mechanism. This assumption is further verified by the absence of droplets for the samples cooled down without As flux (i.e. the As_4_ and indium were closed simultaneously at the end of the growth). Although vapour-liquid-solid (VLS) mechanism has recently been reported in the MBE growth of InAs NWs [28], it is not believed to be the case for our samples. A much higher temperature (530°C) was used for their growths; this would lead to significant As desorption so that the growth was very likely under an indium-rich regime leading to the VLS growth mechanism. However, the indium droplets might lead to growth via VLS in the very early stage due to the presence of indium droplets, e.g. nucleation occurs while both In and As supply and InAs NW growth continues till the excess indium was used up. Then the growth turned to be VS dominant due to the excess of As.

**Figure 2:**
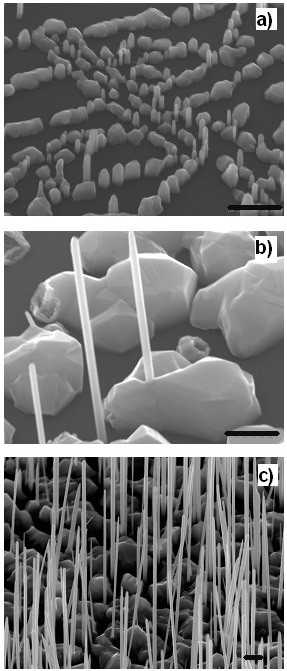
**SEM images tilted at 45° of InAs NWs grown on graphite. (a)** 10, **(b)** 60 and **(c)** 144 min. The scale bar is 500 nm.

**Figure 3:**
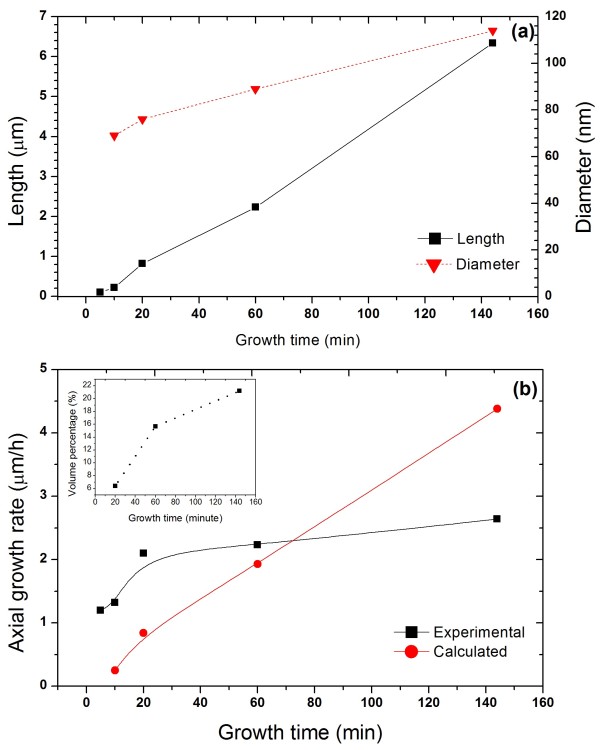
**Measured NWs diameter and length (a) and axial growth rate (b) as function of growth time.** Inset shows the dependence of the ratio of deposited volume between radial and axial growth on growth time.

In order to understand the growth kinetics of NWs on graphite, a series of samples were grown under identical conditions for different growth times. The 45°-tilted SEM images of the resulting samples show that all the growths led to vertically aligned NWs without tapering (see Figure 2). Geometrical parameters of the NWs were deduced from SEM images as shown in Figure 3. We can see that the diameter increases slightly with growth time while the length increases with growth time. Axial growth rate shows two different dependences on growth time, i.e. in the beginning, it increases quickly with growth time then, after 20 min, the rate of increase lessens. This is very different from the dependence observed in the growth of InAs NWs on Si in Ref. [21], where the growth starts with a very fast growth rate which reduces with growth time and saturates at approximately 3 μm h^−1^ after 3 min growth. The difference might be due to the different growth kinetics for the growths on graphite.

The major contributions to the axial growth of NWs include the following [29]: (i) impingement of adatoms on the top of NWs directly, (ii) impingement on the substrate surface and diffusion up the sidewalls, and (iii) impingement on sidewall and diffusion up to the top of NWs. Although this is for VLS growth mechanism, we believe that the principle is applicable to VS growth mode. The major contributors for axial and lateral growths are the adatoms impinging on the surface around NW and on the sidewall of NW. All the adatoms collected from these two sources are finally incorporated into NW growth either through liquid droplet or nucleate directly onto the top of NW, so there is no significant difference between VLS and VS in terms of growth contribution from impinging adatoms. It is well accepted that the contribution from direct impingement on the top of NWs is negligible. The fast increasing growth rate in the beginning is due to the significant contribution from adatoms collected by the surface. With the growth of NWs, more and larger parasitic islands grow on the surface so that the surface area around the NWs collecting incoming adatoms decreases, leading to a reduced contribution from surface collection, and consequently the contribution from sidewall impingement becomes dominant. The axial growth rate, GR, due to the sidewall impingement can be expressed as [21].

$$\mathrm{GR}=\frac{4}{3\sqrt{3}R}L_{\mathrm{diff}}\cos\theta \tan\varphi F_{\mathrm{in}}$$

where *R* is the NW radius, *L*_diff_ is diffusion length along the sidewall, *θ* is the in-plane angle of the normal sidewall with respect to the beam direction, *φ* is the angle of incident beam to the substrate, and *F*_in_ is the nominal growth rate. The value of *θ* varies from 0° to 30° due to hexagonal symmetry of the NWs, *φ* is 30° as defined by our system. Since no tapered NW was observed in our growths, it is obvious that all of the impinging adatoms diffuse along the entire NW length, i.e. the diffusion length is much longer than the length of NWs in our growth. Taking into account the nominal growth rate of 0.1 μm h^−1^, NWs radius of 0.041 μm, and assuming *L*_diff_ > length of NWs *L*, we can estimate the growth rate dependence on *L* as shown in Figure 3b. The radial growth was accounted in the calculation. It can be seen that the experimental growth rate does not follow the calculated dependence. The slower increase of growth rate with growth time can be due to the limitation of the adatoms' diffusion along the sidewall. However, this is not the case in our growths since no tapering is visible. This assumption is consistent to the demonstrations in InAs NWs on Si [21]. Alternatively, we propose that desorption of the adatoms during diffusion along the sidewall plays an important role in the reduced growth rate, which has been reported previously in the growth of NWs on Si [30]. This long diffusion length of the adatoms along the sidewall could be associated to the much slower radial growth rate in comparison with the axial growth rate. Distribution of the overall deposition volume between the radial and axial growth is also shown in inset of Figure 3. It shows that more volume is deposited onto the sidewall with increase of growth time. This is mainly due to the significant increase of the length with increase of growth time; hence, more adatoms could not diffuse up to the tip of NW and contribute to the radial growth.

**Figure 4:**
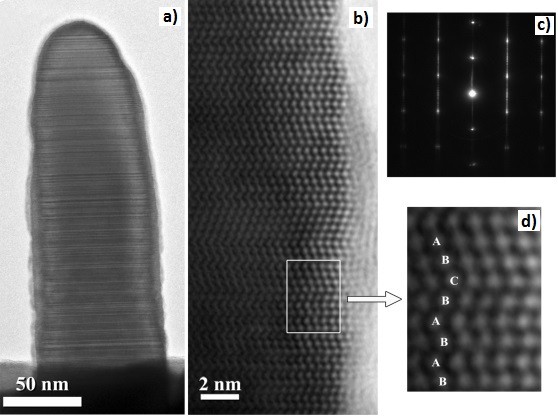
**Images of InAs NW on graphite.** TEM images of an InAs NW on graphite **(a)**; the HRTEM image showing the crystal structure **(b)**; the electron diffraction pattern **(c)** and the enlarged image of the highlighted white rectangular area showing the changes in the stacking **(d)**.

High-resolution TEM (HRTEM) has provided direct experimental evidence of the crystallinity of the InAs nanowires grown on HOPG substrates. The InAs nanowires, with an average diameter of approximately 100 nm, were surrounded by an amorphous layer of a few nanometers thick (see Figure 4a). This amorphous layer is associated with the chemiabsorption of oxygen on the InAs nanowire due to exposure to air [31]. The oxidation of the structure begins with a thin amorphous layer that is observed to form a crystalline phase over time under the electron beam. The NWs grown under these conditions showed a polytype-like structure with mixed wurtzite (WZ) and zinc blende (ZB) character, with multiple stacking faults on (111)/(0001) planes. This polytypism can be easily revealed at higher magnification (Figure 4b). The electron diffraction pattern recorded in similar areas (Figure 4c) shows streaks, indicating the polytype nature of these NWs. The area inside the white rectangle in Figure 4b has been enlarged to highlight the change in the stacking (Figure 4d). The HRTEM inset shows a transition between WZ (BABA) to twinned ZB area (ABCBA). The resulting mixture of crystal structures is similar to previously reported InGaAs NWs grown by MOCVD [2-5]. The ZB phase is normally the most stable crystal structure in bulk III-V semiconductors due to the slightly lower free energy for ZB than that of WZ. However, the crystal structure of materials in nanometer scale is more efficient in reducing the surface energy caused by the large surface-to-volume ratio [32-36]. Theoretical description of the self-catalysed GaAs NWs indicates that WZ phase is thermodynamically favoured for low supersaturation of Ga droplets with As (i.e. low atomic fraction in the Ga droplets), but increase in supersaturation or the shrinkage of the liquid droplets can lead to other phases [37,38]. Thus, III-V NWs with ZB phase are often mixed with WZ phase and related stacking defects such as twin defects, stacking faults and ZB-WZ polytypism.

## Conclusions

In summary, we have demonstrated the MBE growth of InAs NWs on graphite without foreign catalyst, SiO_2_ or patterned substrates. The InAs NWs are vertically aligned on the substrate surface and have a homogeneous diameter distribution without tapering and metal droplets on the tops. Our NWs have a larger diameter, shorter length and less number density in comparison with InAs NWs on Si, which are ascribed to the lack of dangling bond on the graphite surface. The growth was proposed to follow a VS growth mechanism. The surface collection of impinging indium adatoms is the dominant contribution to the axial growth for short NWs, while impinging adatoms on sidewalls and diffusion to the top of the NWs become dominant for the longer NWs. We have also shown that the resulting NWs have mixed pure ZB and WZ insertions.

## Competing interests

The authors declare that they have no competing interests.

## Authors’ contributions

QZ and EA carried out expitaxial synthesis, participated in SEM studies and drafted the manuscript. AS carried out the TEM measurements and analysis. MKR, TDV and AZ carried out SEM measurements. BJR and OK participated in the substrate preparation. VF and FA conceived of the study, and participated in its design and coordination and provided financial support. All authors read and approved the final manuscript.

## References

[B1] Janssen T-J Tzalenchuk A Lara-Avila S Kubatkin S Fal’ko VI Quantum resistance metrology using graphene Rep Prog Phys 2013 9 104501 24088373 10.1088/0034-4885/76/10/104501

[B2] Hoon YJ Lee WH Wu Y Ruoff R Fukui T van der Waals epitaxy of InAs nanowires vertically aligned on single-layer graphene Nano Lett 2012 9 1431 10.1021/nl204109t 22324301

[B3] Hoon YJ Fukui T Controlled van der Waals heteroepitaxy of InAs nanowires on carbon honeycomb lattices ACS Nano 2011 9 7576 10.1021/nn2025786 21838312

[B4] Shin JC Kim KH Yu KJ Hu H Yin L Ning C-Z Rogers JA Zuo J-M Li X In_x_Ga_1‑x_As nanowires on silicon: one-dimensional heterogeneous epitaxy, bandgap engineering, and photovoltaics Nano Lett 2011 9 4831 21967406 10.1021/nl202676b

[B5] Mohseni PK Behnam A Wood JD English CD Lyding JW Pop E Li X In_x_Ga_1−x_As nanowire growth on graphene: van der Waals epitaxy induced phase segregation Nano Lett 2013 9 1153 23421807 10.1021/nl304569d

[B6] Munshi AM Dheeraj DL Fauske VT Kim DC van Helvoort AT Fimland BO Weman H Vertically aligned GaAs nanowires on graphite and few-layer graphene: generic model and epitaxial growth Nano Lett 2012 9 4570 22889019 10.1021/nl3018115

[B7] Kim Y-J Lee J-H Yi G-C Vertically aligned ZnO nanostructures grown on graphene layers Appl Phys Lett 2009 9 213101

[B8] Choi D Choi M-Y Choi WM Shin H-J Park H-K Seo J-S Park J Yoon S-M Chae SJ Lee YH Kim S-W Choi J-Y Lee SY Kim JM Fully rollable transparent nanogenerators based on graphene electrodes Adv Mater 2010 9 2187 20376853 10.1002/adma.200903815

[B9] Chung K Lee C-H Yi G-C Transferable GaN layers grown on ZnO-coated graphene layers for optoelectronic devices Science 2010 9 655 21030653 10.1126/science.1195403

[B10] Zervos M Feiner L-F Electronic structure of piezoelectric double-barrier InAs/InP/InAs/InP/InAs (111) nanowires J Appl Phys 2004 9 281

[B11] Chuang LC Moewe M Chase C Kobayashi NP Chang-Hasnain C Critical diameter for III-V nanowires grown on lattice-mismatched substrates Appl Phys Lett 2007 9 043115

[B12] Ertekin E Greaney PA Chrzan DC Sands TD Equilibrium limits of coherency in strained nanowire heterostructures J Appl Phys 2005 9 114325

[B13] Hu L Chen G Analysis of optical absorption in silicon nanowire arrays for photovoltaic applications Nano Lett 2007 9 3249 17927257 10.1021/nl071018b

[B14] Peng KQ Xu Y Wu Y Yan YJ Lee ST Zhu J Aligned single-crystalline Si nanowire arrays for photovoltaic application Small 2005 9 1062 17193395 10.1002/smll.200500137

[B15] Hua B Motohisa J Kobayashi Y Hara S Fukui T Single GaAs/GaAsP coaxial core − shell nanowire lasers Nano Lett 2009 9 112 19072060 10.1021/nl802636b

[B16] Qian F Gradecak S Li Y Wen CY Lieber CM Core/multishell nanowire heterostructures as multicolor, high-efficiency light-emitting diodes Nano Lett 2005 9 2287 16277469 10.1021/nl051689e

[B17] Czaban JA Thompson DA LaPierre RR GaAs core − shell nanowires for photovoltaic applications Nano Lett 2009 9 148 19143502 10.1021/nl802700u

[B18] Colombo C Heiβ M Gratzel M Fontcuberta i Morral A Gallium arsenide p-i-n radial structures for photovoltaic applications Appl Phys Lett 2009 9 173108

[B19] Wallentin J Anttu N Asoli D Huffman M Åberg I Magnusson MH Siefer G Fuss-Kailuweit P Dimroth F Witzigmann B Xu HQ Samuelson L Deppert K Borgström MT InP nanowire array solar cells achieving 13.8% efficiency by exceeding the ray optics limit Science 2013 9 1057 23328392 10.1126/science.1230969

[B20] Hertenberger S Rudolph D Bolte S Doblinger M Bichler M Spirkoska D Finley JJ Abstreiter G Koblmuller G Absence of vapor-liquid-solid growth during molecular beam epitaxy of self-induced InAs nanowires on Si Appl Phys Lett 2011 9 123114

[B21] Dimakis E Lahnemann J Jahn U Breuer S Hilse M GeeHaar L Riechert H Self-assisted nucleation and vapor–solid growth of InAs nanowires on bare Si(111) Crys Growth Des 2011 9 4001

[B22] Madsen MH Agesen M Krogstrup P Sorensen C Nygard J Influence of the oxide layer for growth of self-assisted InAs nanowires on Si(111) Nanoscale Res Lett 2011 9 516 21880130 10.1186/1556-276X-6-516 PMC3212055

[B23] Jensen LE Bjork MT Jeppesen S Persson AI Ohlsson BJ Samuelson L Role of surface diffusion in chemical beam epitaxy of InAs nanowires Nano Lett 2004 9 1961

[B24] Murakami S Funayama H Shimomura K Waho T Au-assisted growth of InAs nanowires on GaAs(111)B, GaAs(100), InP(111)B, InP(100) by MOVPE Phys Status Solidi C 2013 9 761

[B25] Mandl B Stangl J Mårtensson T Mikkelsen A Eriksson J Karlsson LS Bauer GU Samuelson L Seifert W Au-free epitaxial growth of InAs nanowires Nano Lett 2006 9 1817 16895379 10.1021/nl060452v

[B26] Koblmuller G Hertenberger S Vizbaras K Bichler M Bao F Zhang J-P Abstreiter G Self-induced growth of vertical free-standing InAs nanowires on Si(111) by molecular beam epitaxy Nanotechnology 2010 9 365602 20702932 10.1088/0957-4484/21/36/365602

[B27] Dubrovskii VG Cirlin GE Soshnikov IP Tonkikh AA Sibirev NV Samsonenko YB Ustinov VM Diffusion-induced growth of GaAs nanowhiskers during molecular beam epitaxy: theory and experiment Phys Rev B 2005 9 205325

[B28] Th G Rieger T Ch B Th S Grützmacher D Lepsa MI Self-catalyzed VLS grown InAs nanowires with twinning superlattices Nanotechnology 2013 9 335601 10.1088/0957-4484/24/33/335601 23881182

[B29] Harmand J-C Glas F Patriarche G Growth kinetics of a single InP_1−x_As_x_ nanowire Phys Rev B 2010 9 235436

[B30] Colombo C Spirkoska D Frimmer M Abstreiter G Fontcuberta i Morral A Ga-assisted catalyst-free growth mechanism of GaAs nanowires by molecular beam epitaxy Phys Rev B 2008 9 155326

[B31] Werner F Limbach F Carsten M Denker C Malindretos J Rizzi A Electrical conductivity of InN nanowires and the influence of the native indium oxide formed at their surface Nano Lett 2009 9 1567 19290610 10.1021/nl8036799

[B32] Glas F Harmand J-C Patriarche G Why does wurtzite form in nanowires of III-V zinc blende semiconductors? Phys Rev Lett 2007 9 146101 17930689 10.1103/PhysRevLett.99.146101

[B33] Dick KA Caroff P Bolinsson J Messing ME Johansson J Deppert K Wallenberg LR Samuelson L Control of III–V nanowire crystal structure by growth parameter tuning Semicond Sci Technol 2010 9 024009

[B34] Johansson J Dick KA Caroff P Messing ME Bolinsson J Deppert K Samuelson L Diameter Dependence of the wurtzite-zinc blende transition in InAs nanowires J Phys Chem C 2010 9 3837

[B35] Yamashita T Akiyama T Nakamura K Ito T Theoretical investigation on the structural stability of GaAs nanowires with two different types of facets Phys E 2010 9 2727

[B36] Akiyama T Sano K Nakamura K Ito T An empirical potential approach to wurtzite–zinc-blende polytypism in group III–V semiconductor nanowires J J Appl Phys 2006 9 L275

[B37] Krogstrup P Popovitz-Biro R Johnson E Hannibal Madsen M Nygård J Shtrikman H Structural phase control in self-catalyzed growth of GaAs nanowires on silicon (111) Nano Lett 2010 9 4475 20932012 10.1021/nl102308k

[B38] Krogstrup P Curiotto S Johnson E Aagesen M Nygård J Chatain D Impact of the liquid phase shape on the structure of III-V nanowires Phys Rev Lett 2011 9 125505 21517326 10.1103/PhysRevLett.106.125505

